# Imaging findings of lower limb involvement following COVID-19

**DOI:** 10.1259/bjrcr.20210219

**Published:** 2022-09-12

**Authors:** Ivan Rodrigues Barros Godoy, Tatiane Cantarelli Rodrigues, Gabriela dos Santos Zamariolli, Gustavo Pimenta de Figueiredo Dias, André Fukunishi Yamada, Abdalla Youssef Skaf

**Affiliations:** 1 Department of Radiology, Hospital do Coração (HCor) and Teleimagem, São Paulo, Brazil; 2 Department of Diagnostic Imaging, Federal University of São Paulo (UNIFESP), São Paulo, Brazil; 3 ALTA Diagnostic Center (DASA Group), São Paulo, Brazil

## Abstract

Coronavirus disease 2019 (COVID-19) is known mainly by the severe acute respiratory syndrome, with myalgia as a common clinical symptom. Recent reports described musculoskeletal complications related to COVID-19 such as myositis, neuropathy and arthropathy.

Radiologists and ordering physicians should be aware of lower limb complications following severe COVID-19 for optimal patient care.

## Summary

There have been recent reports of musculoskeletal complications related to COVID-19 including myositis; neuropathy; arthropathy including osteonecrosis, joint effusion and synovitis; vascular thrombosis and other soft tissue abnormalities such as heterotopic ossification. Imaging studies are important to evaluate extrapulmonary involvement in COVID-19 patients.^1–4^ We describe imaging findings in a series of patients with lower limb musculoskeletal manifestations after COVID-19.

Imaging findings suspicious for musculoskeletal involvement related to COVID-19 are being increasingly reported.^4,5^ Numerous patients presented with muscle edema in case reports due to myositis and rhabdomyolysis as a presenting symptom or late complication.^3,5,6^ Effects of COVID-19 in the musculoskeletal system are not completely comprehended and may be due to hematogenous spread and muscle cell viral invasion through the ACE2 receptor have recently been suggested.^7,8^ Cytokine storm and activation of immune-mediated mechanisms are also an alternate concept of muscle involvement of SARS-CoV-2.^7,8^

## Myositis

### Ankle and foot myopathy

**Figure 1.**
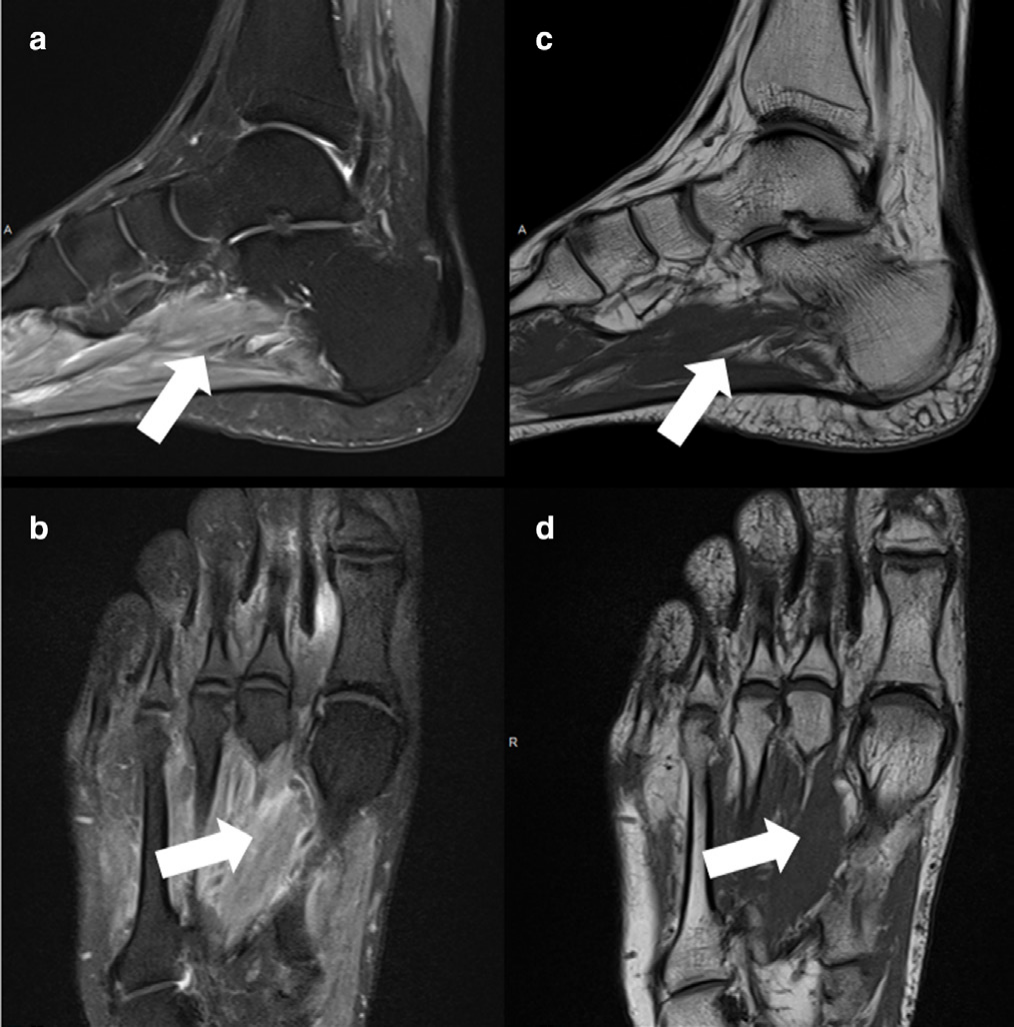
Foot MR. (**A**) Sagittal and (**B**) axial T2 fat-saturated imaging demonstrated plantar and interosseous muscle edema, without significant fatty atrophy on (**C**) sagittal and (**D**) axial T1 (white arrows).

A 43-year-old male previously hospitalized for COVID-19 with mechanical ventilation and treated with injectable corticosteroids, with foot and ankle pain and edema 4 months after the COVID-19 diagnosis. Magnetic resonance (MR) imaging of the ankle and foot demonstrated plantar and interosseous muscle edema, without significant fatty atrophy (Figure 1).

### Leg myopathy

**Figure 2.**
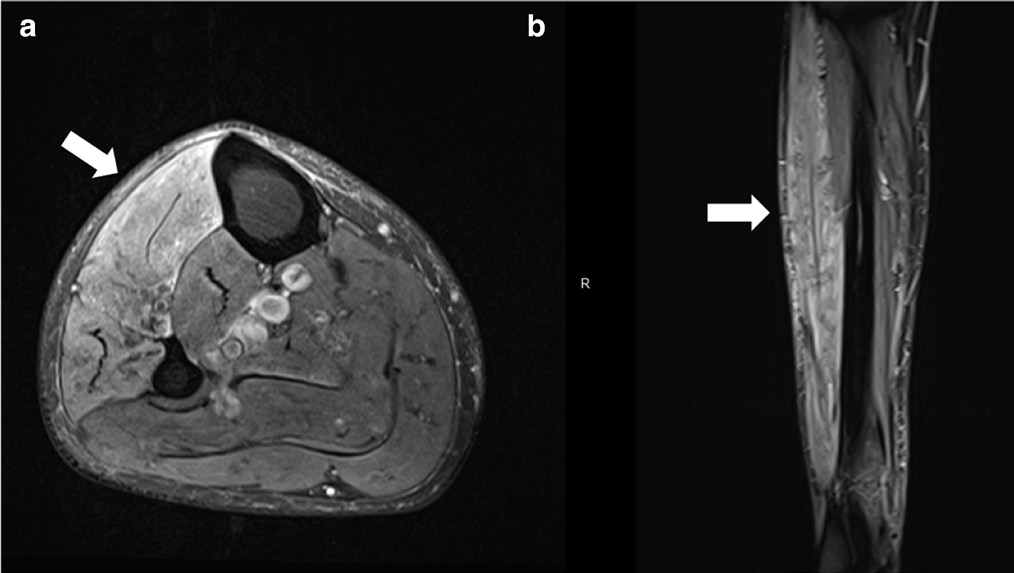
Leg MR imaging demonstrated (**A**) axial and (**B**) coronal T2 fat-saturated diffuse muscle edema, involving the anterolateral muscular compartment of the leg.

A 75-year-old male previously hospitalized for COVID-19 without mechanical ventilation, with leg pain and weakness 3 months after COVID-19 diagnosis. Leg MR imaging demonstrated diffuse muscle edema, involving the anterolateral muscular compartment of the leg (Figure 2).

### Pelvis myopathy

**Figure 3.**
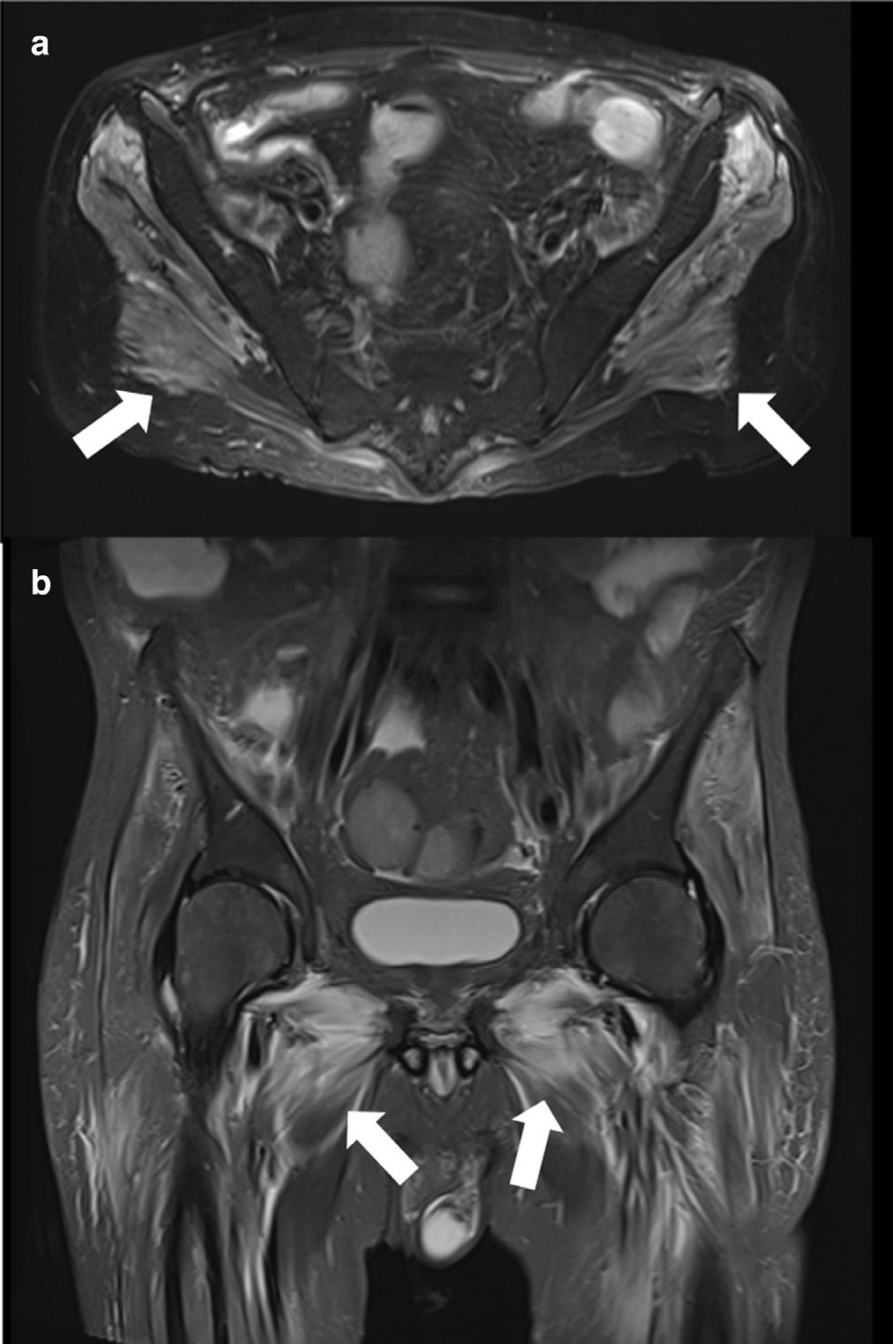
Pelvis MR. (**A**) Sagittal and (**B**) axial T2 fat-saturated imaging demonstrated diffuse muscle edema involving bilateral adductor and gluteus muscles.

A 48-year-old male previously hospitalized for COVID-19 with mechanical ventilation and treated with injectable corticosteroids, with pelvis pain, weakness and edema 4 months after the COVID-19 diagnosis. MR imaging demonstrated diffuse muscle edema involving bilateral adductor and gluteus muscles (Figure 3).

## Discussion

Numerous patients presented with muscle edema in case reports due to myositis and rhabdomyolysis as a presenting symptom or late complication.^3,5,6^ Recent reports have proposed theories to understand muscular involvement in COVID-19 such as hematogenous spread and direct viral invasion of skeletal muscle cells through the ACE2 receptors.^7,8^ Also, it has been suggested that COVID-19 infection may provoke an immune-mediated muscle injury due to an inflammatory response with cytokine storm, immune cells activation and deposition of immune complex with myotoxic effect.^7,8^ Muscular edema on MRI also be observed in COVID-19 patients with critical illness myopathy, that is usually associated in intensive care unit patients and also with corticosteroid use and are distinctive from other causes of myositis in imaging. Myonecrosis and rhabdomyolysis are not present in critical illness myopathy, and may be helpful for diagnosis.^4^

### Learning points

1. Myositis may be a complication following COVID-19 infection, MR imaging is the preferred method to identify and evaluate the extension of muscle edema.
2. Follow-up imaging may be performed to evaluate muscle edema resolution or fatty atrophy.
3. Extensive and multicompartmental muscle edema should be concerned for rhabdomyolysis.

## Osteonecrosis

### Knee osteonecrosis

**Figure 4.**
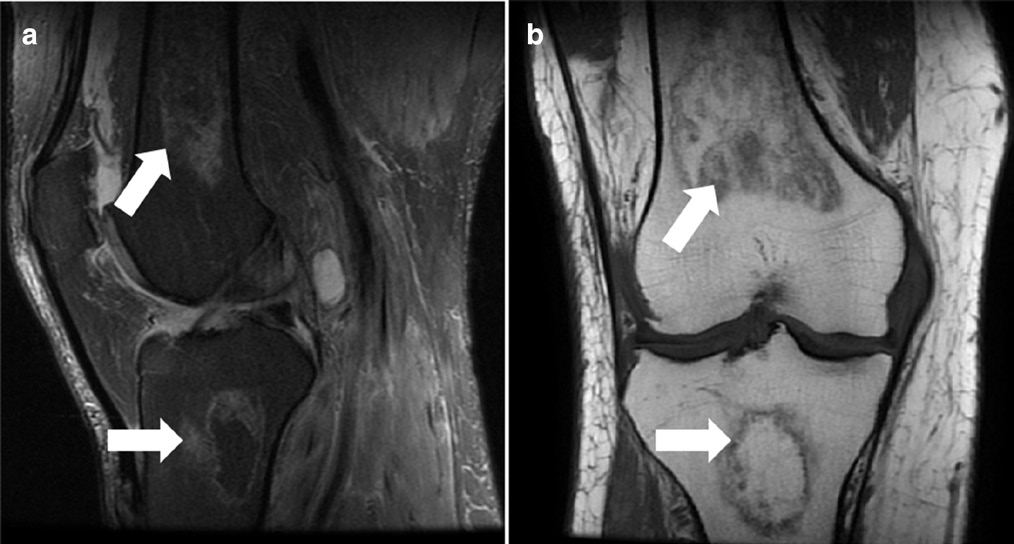
Knee MR. (**A**) Sagittal T2 fat-saturated and (**B**) coronal imaging osteonecrosis in distal femur and proximal tibial (white arrows).

A 57-year-old male previously hospitalized for COVID-19 with mechanical ventilation and treated with injectable corticosteroids, with knee pain and edema for 6 months after the COVID-19 diagnosis. MR imaging demonstrating osteonecrosis in distal femur and proximal tibia (Figure 4).

### Ankle osteonecrosis

**Figure 5.**
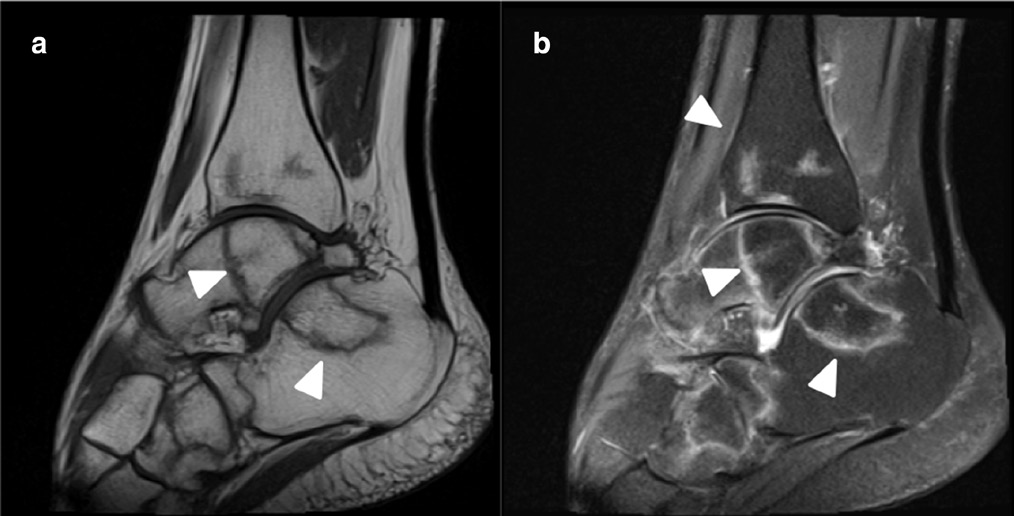
Ankle MR. (**A**) Sagittal T2 fat-saturated and (**B**) coronal imaging osteonecrosis in distal tibia, talus, calcaneus and cuboid (white arrows).

A 28-year-old male previously hospitalized for COVID-19 without mechanical ventilation and treated with injectable corticosteroids, with ankle pain and edema for 2 months after the COVID-19 diagnosis. Ankle MR imaging demonstrates osteonecrosis in distal tibia, talus, calcaneus and cuboid (Figure 5).

## Discussion

Currently, there are few reports of osseous complications of COVID-19 and it is still unclear if the development of osteoporosis and osteonecrosis is mainly caused by virus-induced coagulopathy or by effects of critical illness and corticosteroid use.^9,10^ Imaging studies may demonstrate serpiginous sclerosis on CT and radiography, and irregular line with adjacent bone marrow edema on MR imaging. In late stages of osteonecrosis, articular surface collapse and joint effusion may be present.

## Learning points

1. MR imaging is helpful to identify bone abnormalities related to COVID-19 infection. Post-treatment changes such as corticosteroid use also should be considered as cause of osteonecrosis in COVID-19 patients.
2. CT and radiography may be normal in early stages of osteonecrosis.

## Heterotopic ossification

### Hip heterotopic ossification

**Figure 6.**
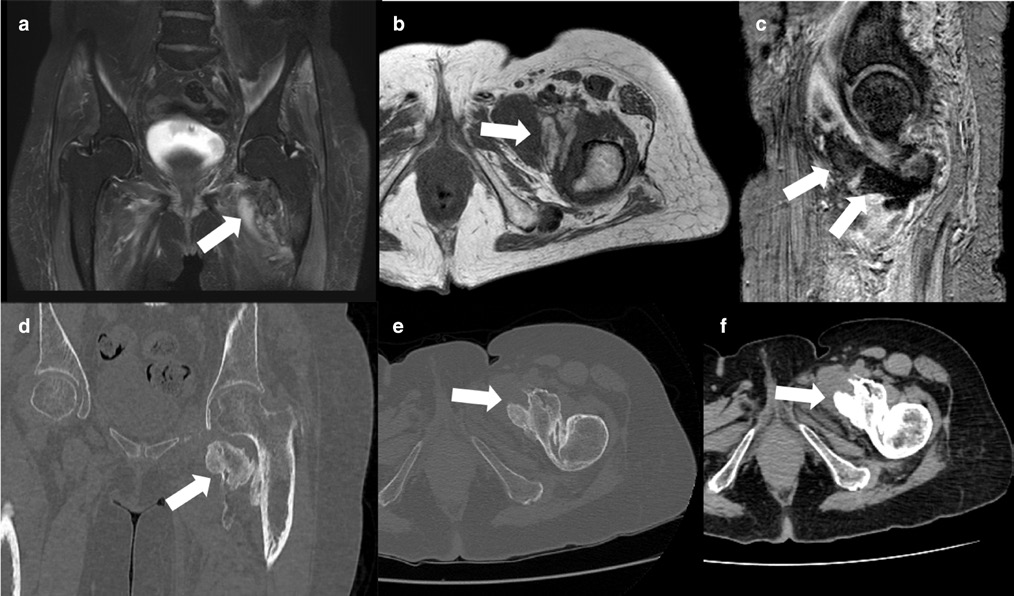
Hip MR. (**A**) Coronal T2 fat-saturated, and (**B**) axial T1 and (**C**) sagittal GRE imaging demonstrated anterior periarticular heterotopic ossification. Left hip CT coronal (**D**) axial (**E**) and axial soft-tissue window (**F**) images showing a large anterior periarticular heterotopic ossification. GRE, gradient-echo.

A 68-year-old male previously hospitalized for COVID-19 with mechanical ventilation and treated with injectable corticosteroids, with hip pain, edema and movement deficit 2 months after the COVID-19 diagnosis. MR imaging demonstrated anterior periarticular heterotopic ossification (Figure 6).

## Discussion

Recent reports described heterotopic ossification following severe COVID-19 infection, and may be related to long-lasting hospitalization with immobilization and hypoxia.^11^ The prevalence of heterotopic ossification in COVID-19 patients was reported as four times higher than in patients with other causes of acute respiratory distress syndrome, and could be related to other factors such as altered calcium metabolism in critical illness and myositis secondary to the viral infection.

## Learning points

1. Periarticular heterotopic ossification should be suspected in a patient with reduced range of movement with history of immobilization and long-term hospitalization.

## Thrombosis

### Arterial thrombosis

**Figure 7.**
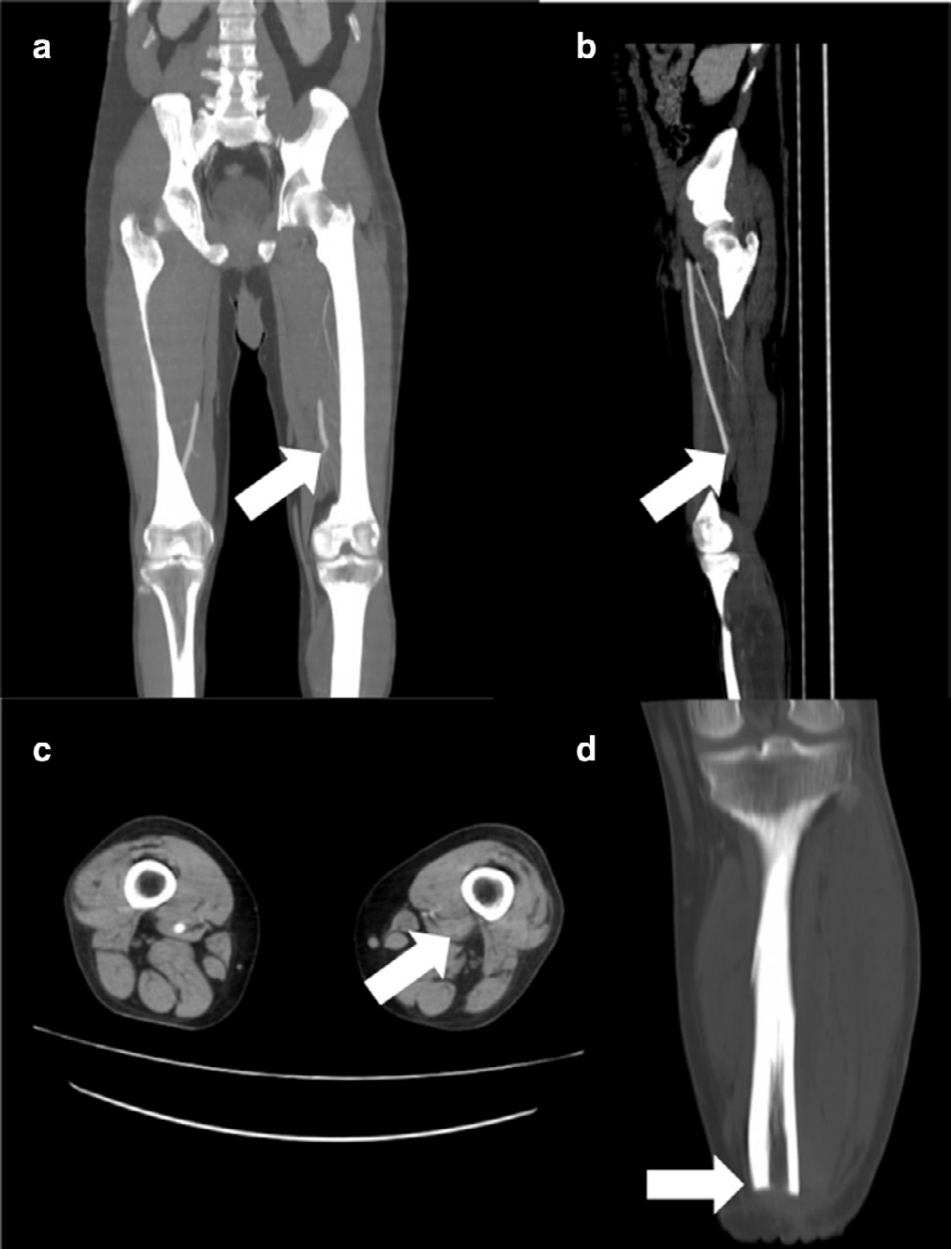
(**A**) Coronal, (**B**) sagittal and (**C**) axial post-contrast CT images showing absence of the intravascular contrast in the left arterial femoral-popliteal segment compatible with thrombosis. (**D**) Coronal CT image showing post-treatment changes after amputation.

A 59-year-old male previously hospitalized for COVID-19 with mechanical ventilation and treated with injectable corticosteroids, with intense leg pain 2 months after the COVID-19 diagnosis. Post-contrast CT images showing absence of the intravascular contrast in the left arterial femoral-popliteal segment compatible with thrombosis. Coronal CT image showing post-treatment changes after amputation (Figure 7).

## Discussion

COVID-19 is also known for coagulopathy due to viral infection and may induce thrombosis and thromboembolic events.^9,10^ Also, there are reports of disseminated intravascular coagulation and gangrene, due to thromboembolic events and also therapy with vasopressors for hemodynamic support in severe COVID-19 cases.^13^

## Learning points

1. Thrombotic events are common in COVID-19 infection and imaging studies such as ultrasound and post-contrast CT or MR imaging may be used for diagnosis and follow-up.

## Conclusions

In conclusion, COVID-19-related lower limb conditions may require a multimodality imaging approach using radiography, CT, ultrasound and MR imaging for diagnosis and therapy guidance.. Awareness of lower limb musculoskeletal involvement may help minimize functional impairment, especially after severe cases that needed ICU with mechanical ventilation, long period of sedation and immobilization due to neuromuscular blockade and also with corticosteroid use.
